# Sensitivity analysis of an asymmetric Monte Carlo beam model of a Siemens PRIMUS accelerator

**DOI:** 10.1120/jacmp.v13i2.3402

**Published:** 2012-03-08

**Authors:** Eric C. Schreiber, Daren L. Sawkey, Bruce A. Faddegon

**Affiliations:** ^1^ Department of Radiation Oncology University of North Carolina at Chapel Hill Chapel Hill North Carolina 27514; ^2^ Department of Radiation Oncology University of California San Francisco San Francisco California 94143 USA

**Keywords:** Monte Carlo, accelerator modeling, beam asymmetry

## Abstract

The assumption of cylindrical symmetry in radiotherapy accelerator models can pose a challenge for precise Monte Carlo modeling. This assumption makes it difficult to account for measured asymmetries in clinical dose distributions. We have performed a sensitivity study examining the effect of varying symmetric and asymmetric beam and geometric parameters of a Monte Carlo model for a Siemens PRIMUS accelerator. The accelerator and dose output were simulated using modified versions of BEAMnrc and DOSXYZnrc that allow lateral offsets of accelerator components and lateral and angular offsets for the incident electron beam. Dose distributions were studied for $40\times 40\;{\text{cm}}^{2}$ fields. The resulting dose distributions were analyzed for changes in flatness, symmetry, and off‐axis ratio (OAR). The electron beam parameters having the greatest effect on the resulting dose distributions were found to be electron energy and angle of incidence, as high as 5% for a 0.25° deflection. Electron spot size and lateral offset of the electron beam were found to have a smaller impact. Variations in photon target thickness were found to have a small effect. Small lateral offsets of the flattening filter caused significant variation to the OAR. In general, the greatest sensitivity to accelerator parameters could be observed for higher energies and off‐axis ratios closer to the central axis. Lateral and angular offsets of beam and accelerator components have strong effects on dose distributions, and should be included in any high‐accuracy beam model.

PACS numbers: 87.55.K‐, 87.55.Gh

## I. INTRODUCTION

Monte Carlo plays an increasingly important role in clinical dosimetry calculations. While several commercially available Monte Carlo codes contain mathematical accelerator models, such as the two‐source model used in the CyberKnife treatment planning system,^(1)^ performing radiotherapy Monte Carlo calculations to 1%/1 mm accuracy likely requires an explicit simulation of each component in the treatment head, including the components' location, dimensions, and material composition. A fully geometric rendering of a linear accelerator represents a very large number of adjustable parameters in a model. Accelerator specifications supplied by the manufacturer provide a good starting point for developing Monte Carlo models, but these cannot account for deviations from factory specifications. Source information provided is often limited to nominal energy and spot size, and customization of the Monte Carlo model is generally required. The reliance of Monte Carlo simulation as a realistic physical description of the accelerator advises that modifications to the accelerator model intended to “tweak” simulation results to match experimental data should correspond to actual physical deviations from the initial model. These deviations are, in most cases, difficult or impossible to measure, and so must be inferred from measureable beam data. Owing in part to the large number of adjustable parameters, commissioning a Monte Carlo linear accelerator simulation can be a more rigorous and difficult process than it is for more standard treatment planning software.

Most radiotherapy computational models, including Monte Carlo, assume that non‐beam–shaping accelerator components are cylindrically symmetric and aligned along the central axis. This assumption makes the geometry of the accelerator components easier to define, but does not always reflect the true configuration of a real accelerator.^(^ ^2^ ^,^ ^3^ ^)^ The most obvious example of this is the small asymmetry in the in‐plane direction attributed to a small angular deviation from the central axis of the electron beam as it emerges from the bending magnet. Other possible sources of asymmetry include lateral offsets of the electron beam and the flattening filter.^(4)^ While well‐tuned clinical beams generally exhibit only small asymmetries (<2%), achieving 1%/1 mm modeling accuracy may require accounting for even these small deviations. In order to obtain the most accurate simulation results, the potential for asymmetric linac head components and off‐axis electron source configurations should be included in the Monte Carlo commissioning process.

Correlating the many adjustable simulation parameters with measurable dose distributions can greatly facilitate the commissioning process. Monte Carlo sensitivity studies are an effective means of relating treatment head parameters to clinical measurements. Bieda et al.^(5)^ studied the effect of scattering foil parameters on electron dose distributions. This work was expanded by Weinberg et al.^(6)^ to include variations in the electron source parameters for large electron fields. Schreiber and Faddegon^(2)^ also performed a sensitivity study for large electron fields, including the effect of beam model asymmetries on the resulting dose distributions. The sensitivity of photons beams to electron and linear accelerator parameters has been studied by Sheikh‐Bagheri and Rogers.^(7)^

We have performed a sensitivity analysis for a Siemens PRIMUS accelerator, in which small variations of linear accelerator and electron source parameters are characterized by their impact on the shape and symmetry of the resulting dose profiles in a water phantom. The focus is configurations leading to asymmetric dose distributions, although some symmetric accelerator structures have been included in the study, as well. The intention of this study is to provide guidance on the correlation between accelerator parameters and measured dose profiles to two groups: users creating highly‐accurate Monte Carlo beam models of their linacs for clinical and research purposes, and clinical physicists needing to physically adjust a linear accelerator to bring beam parameters to within clinically acceptable levels. The accuracy of the accelerator simulation for a clinical accelerator with source and geometry asymmetry has been experimentally validated at 6 MV and 18 MV nominal X‐ray beam energies with direct measurement.^(^ ^3^ ^,^ ^4^ ^,^ ^8^ ^)^ The Monte Carlo code itself has been thoroughly benchmarked with measured data, and is among the most accurate codes for simulation of linac treatment heads used in radiotherapy (see, for example, Faddegon et al.^(9)^). The results of the sensitivity analysis, relating changes in source and geometry details to changes in dose distributions, are at least as accurate as the clinical beam comparison and experimental benchmarks.

## II. MATERIALS AND METHODS

**Figure 1:**
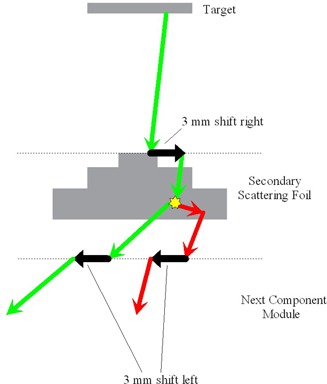
Schematic representation of modifications to BEAMnrc allowing lateral shifts of accelerator components.

**Figure 2:**
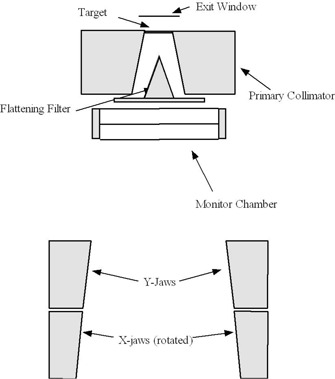
Schematic of Siemens PRIMUS accelerator simulation.

The sensitivity study was performed using the EGSnrc‐based radiation transport software packages BEAMnrc^(10)^ and DOSXYZnrc.^(11)^ Radiation transport through the linear accelerator was simulated using a modified version of BEAMnrc. Routines were added to BEAMnrc that enabled lateral offsets of component modules (Fig. 1) and lateral and angular offsets of electrons beams in BEAMnrc source models. The linac model, shown schematically in Fig. 2, included the exit window, photon target, primary collimator, flattening filter, monitor chamber, Y jaws, and multileaf collimator (MLC). Note that in a PRIMUS accelerator, the MLC serves as the X jaws. The radiation transport parameters used are shown in Table 1. The only variance reduction technique used was bremsstrahlung splitting in the photon target, in which each photon‐producing electron interaction in the target was repeated 100 times. The BEAMnrc simulation was compiled as a library to be used as a source model for DOSXYZnrc.

**Table 1:** Summary of EGSnrc parameters.

*Parameter*	*Value*
Global ECUT	0.700
Global PCUT	0.01
Global SMAX	5.0
ESTEPE	0.25
XIMAX	0.5
Boundary crossing algorithm	PRESTA‐I
Skin depth for BCA	0
Electron‐step algorithm	PRESTA‐II
Spin effects	On
Bremsstrahlung angular sampling	Simple
Bremsstrahlung cross sections	Bethe‐Heitler
Bound Compton scattering	Off
Pair angular sampling	Simple
Photoelectric angular sampling	Off
Raleigh scattering	Off
Atomic relaxations	Off
Electron impact ionization	Off

The BEAMnrc linear accelerator model was based on a Siemens PRIMUS accelerator (Siemens Medical Solutions, Malvern, PA) using specifications obtained from the manufacturer as a starting point. These specifications were verified by careful measurements performed on a clinical accelerator of the same model that was dedicated to research.^(4)^ We studied the accelerator operating in X‐ray mode at 6 and 18 MV with a 100 cm source‐to‐surface distance (SSD). For all simulations, the jaws and multileaf collimators were fully opened to form a 40cm×40cm field. The large field was selected to maximize the sensitivity of off‐axis dose to the treatment head parameters. The relative ease in modeling beam‐shaping elements allows a straightforward recovery of clinically relevant beams from beam models derived from large‐field simulations. Because the treatment parameters studied are located in the interior of the field, a detailed model of MLC leakage was not necessary, so the MLC was modeled as a solid jaw. Sample simulations using an explicit MLC model were found to produce dose distributions negligibly different from those where the MLC was modeled as a jaw.

Previous work has demonstrated that removing the flattening filter from a linear accelerator can provide potential clinical benefits^(12)^ and/or simplify the measurement of electron source parameters.^(4)^ Therefore, in addition to the full accelerator model used, additional simulations were performed for the accelerator with the flattening filter removed.

Radiation transport in the water phantom was simulated using DOSXYZnrc. The simulated water phantom was 40 cm deep, 80 cm in length and width, and comprised of 2cm×2cm×0.5cm voxels. For studies of asymmetric parameters, the voxel size was reduced to 1 cm in the direction of the asymmetry. Simulations were calculated to a statistical uncertainty of 0.5% or better in the high‐dose region.

Accelerator parameters involved in the sensitivity study were divided into those producing symmetric and asymmetric variations in the dose distribution. Symmetric variations are defined as those producing changes in the dose profile in flatness and OAR, but leaving beam symmetry unchanged. Symmetric variations of electron source parameters included in the study include electron energy and energy distribution, electron divergence angle, and electron spot size. Photon target thickness was included as a symmetric variation of the linear accelerator model. Asymmetric variations are defined as those producing changes in dose profile symmetry, as well as flatness and OAR. Asymmetric variations are the result of a relative lateral or angular offset between the central axis of the electron source and the flattening filter. Asymmetric parameters included in the study include electron beam angle, lateral offset of the electron beam, and lateral offset of the flattening filter. A summary of all parameters studied is shown in Table 2.

**Table 2:** Model parameters varied.

*Parameter*	*Range*
Electron Source Parameters:	
Average Energy	±9%
Energy spread (FWHM)	0–20%
Spot size	0–4mm
Angular divergence	$0–1^{\circ }$
Beam angle	$0–1^{\circ }$
Offset from central axis	0–2mm
Linear Accelerator Parameters:	
Target thickness	±10%
Flattening filter offset from central axis	0–2 mm

Dose results were analyzed by extracting the percentage depth dose (PDD), and in‐plane and cross‐plane profiles at $D_{\max}$ (1.5 cm for 6 MV, 3.0 cm for 18 MV), 10 cm depth, and 20 cm depth. The profiles were analyzed for area symmetry and changes in off‐axis ratio (OAR). Symmetry was defined as (U−V)/(U+V), where *U* is the integration of the dose values from the left 50% dose level to the central axis, and *V* is the corresponding integration on the right side. OAR was analyzed near the central axis (5 cm) and farther from the axis (15 cm). Profile flatness was found to be less sensitive than OAR without providing new information, and is not included in this report.

## II. RESULTS & DISCUSSION

Results for asymmetric beam variations are quantified by the changes in beam symmetry, as shown in Table 3. Results for symmetric beam variations are tabulated as changes in OAR, shown in Table 4 and Table 5. The results are stated as the degree of change in a given parameter required to adjust a specific result by 2%. The value for the 2% shift was determined by fitting a trend line to the data over the full range tested. The 2% standard was chosen to put the results in a form most useful for modeling well‐tuned clinical accelerators, or for representing the likely magnitude of adjustments required to bring an accelerator to within acceptable specifications.

**Table 3:** Parameter changes required to create a 2% change in symmetry.

	*Adjustment for 2% Change in Symmetry*		
	$d_{\text{max}}$	Depth=10 cm	Depth=20 cm
6 MV	$\left(d_{\max}=1.5\;\text{cm}\right)$		
Electron beam deflection	0.2 deg	0.2 deg	0.2 deg
Electron beam offset	0.7 mm	0.6 mm	0.6 mm
Flattening filter offset	0.6 mm	0.6 mm	0.7 mm
18 MV	$\left(d_{\max}=3.2\;\text{cm}\right)$		
Electron beam deflection	0.1 deg	0.1 deg	0.1 deg
Electron beam offset	0.3 mm	0.3 mm	0.3 mm
Flattening filter offset	0.2 mm	0.2 mm	0.3 mm
6MV – No FF	$\left(d_{\text{max}}=1.5\text{cm}\right)$		
Electron beam deflection	0.4 deg	0.3 deg	0.3 deg
18 MV – No FF	$\left(d_{\max}=3.2\;\text{cm}\right)$		
Electron beam deflection	0.1 deg	0.1 deg	0.1 deg

**Table 4:** Parameter changes required to adjust OAR 2% for 6 MV configuration.

		OAR–6 MV
	*5 cm*	*15 cm*
Energy Change			
$d_{\max}(1.5\;\text{cm})$	3.3%	10.5%
Depth=10 cm	4.0%	10.0%
Depth=20 cm	4.3%	14.3%
Spot Size		
$d_{\max}(1.5\;\text{cm})$	5.7 mm	15.4 mm
Depth=10 cm	3.3 mm	4.3 mm
Depth=20 cm	13.3 mm	25 mm
Target Thickness		
$d_{\max}(1.5\;\text{cm})$	100%	200%
Depth=10 cm	40%	200%
Depth=20 cm	500%	250%

**Table 5:** Parameter changes required to adjust OAR 2% for 18 MV configuration.

		OAR–18 MV
	*5 cm*	*15 cm*
Energy Change		
$d_{\max}(3.2\;\text{cm})$	2.6%	6.1%
Depth=10 cm	2.8%	6.5%
Depth=20 cm	3.3%	7.1%
Spot Size		
$d_{\max}(3.2\;\text{cm})$	5.0 mm	5.0 mm
Depth=10 cm	4.0 mm	10.0 mm
Depth=20 cm	4.4 mm	8.2 mm
Target Thickness		
$d_{\max}(3.2\;\text{cm})$	22%	200%
Depth=10 cm	22%	100%
Depth=20 cm	28%	280%

**Figure 3:**
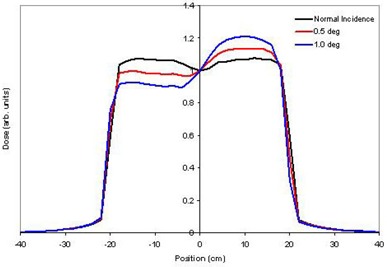
Change in 6 MV $d_{\text{max}}$ profile for small changes in electron beam angle.

**Figure 4:**
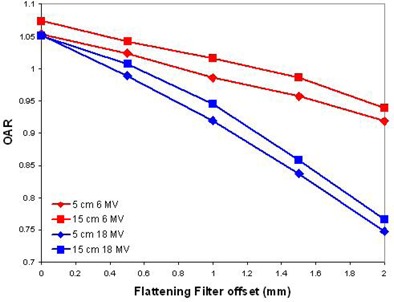
Linear trend line for beam symmetry vs. electron angle of incidence.

Changes in the symmetry of dose profiles correlated with configurations representing a lack of alignment between the central axis of the electron beam and the central axis of the flattening filter. The incident angle of the electron beam had a large effect on the dose profiles (Fig. 3). A 0.25° deviation from normal was found to change the symmetry by 2.1% for 6 MV and 5.5% for 18 MV (Fig. 4). Small lateral offsets to the flattening filter had a large effect on the dose profiles. For a given lateral offset, the off‐axis ratio was more strongly affected close to the central beam axis (5 cm) than at greater radii (15 cm) for both energies studied. This is likely due to the greater slope of the flattening filter near its central axis. Lateral offsets of the incident electron beam produced results that were indistinguishable from flattening filter offsets. This is as expected, as both offsets reflect the same relative misalignment between the two central axes of the electron beam and flattening filter. The electron source and flattening filter must be considered as a system to evaluate asymmetries in the dose profiles.

Altering the electron beam energy had a strong effect on the OAR. The thickness of the primary target had a comparatively minor effect on the dose distributions. Varying the electron spot size had only a small effect on the dose distributions, affecting flatness approximately 0.5% for 6 MV and negligibly for 18 M V. Variations of the electron energy distribution (0%–20%) and divergence angle (0–1.0 degree) had no observable effect. These results are consistent with previous studies of Siemens accelerators,^(7)^ with the small differences likely due to variations between the two accelerator models used.

The sensitivity analysis shows the difficulties inherent in commissioning a Monte Carlo treatment head simulation, in that multiple parameter changes can seem to produce the same alteration in measureable dose distributions. However, several trends are apparent which can direct a physicist undertaking a commissioning task to the areas of greatest sensitivity.

**Figure 5:**
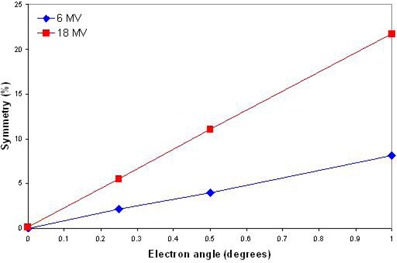
Off‐axis ratio versus lateral offset of flattening filter. OAR's 5 and 15 cm from the CAX are considered.

One trend is that high‐energy configurations tend to be more sensitive to small parameter changes than lower energy configurations. For example, the flattening filter lateral offset required to induce a 2% change in symmetry is roughly three times greater for the 6 MV configuration than for the 18 MV configuration (Fig. 5, Table 3). This observation is obviously most useful for components shared between high‐ and low‐energy configurations. In a Siemens PRIMUS accelerator, the treatment head parameters most responsible for dose profile asymmetries (electron beam parameters and flattening filter position and design) are not shared between configurations.

A second observation is that the OAR is more sensitive near the central axis than in the periphery of the field. For example, the OAR 5 cm from the central axis can change significantly for a 10%–20% change in target thickness, but an unrealistic change in target thickness is required to significantly alter the OAR at 15 cm (Tables 4and 5). Similarly, 2% changes in OAR at 5 cm require roughly half as large a change in electron beam energy as the OAR at 15 cm.

Finally, lateral offsets of the flattening filter and electron beam, and, to a lesser extent, angular offsets of the electron beam, produce similar changes in beam symmetry and can be difficult to untangle. This can be overcome by measuring dose profiles with the flattening filter removed.^(^ ^4^ ^,^ ^13^ ^)^ In this configuration, all dose asymmetries are determined by electron beam parameters. The differing effects of electron incident angle and lateral offset can be separated by noting the change in the lateral position of the dose distribution peak versus depth. While removing the flattening filter can be impractical for a linear accelerator already in clinical use, measurements performed during acceptance testing of a new accelerator may be possible and could simplify later Monte Carlo commissioning work.

This work is primarily intended to facilitate the commissioning of highly accurate (1%/1 mm) Monte Carlo models of clinical accelerators. The results are useful for determining a combination of asymmetries for a given linac — some of these constrained by measurement — to match the measured beam symmetry. The results also provide quantitative physical reasoning to physicists or technicians needing to adjust an accelerator to correct dose profiles to within a clinically acceptable range. This includes source and geometry adjustments made to newly installed linacs, to linacs having undergone a major service, and to a commissioned linac in clinical service. Commissioned linacs are known to develop asymmetric X‐ray beams on occasion, for example, through a change in the beam steering or an error in flattener positioning from a mechanical fault.

## IV. CONCLUSIONS

This study demonstrates the importance of including asymmetric components to beam models, as small deviations from cylindrical symmetry were found to have large effects in the dose distribution. Consequently, the smaller asymmetries found in clinical commissioning data would correspond to system asymmetries too small to easily measure. The tabulated results of this study can be a useful tool when commissioning an asymmetric Monte Carlo beam model, giving the physicist helpful insight into what parameters can be changed to match simulations with measured results.

## ACKNOWLEDGMENTS

This work was supported in part by NIH Grant No. R01 CA104777‐01A2
